# Ergosterol Peroxide Disrupts Triple-Negative Breast Cancer Mitochondrial Function and Inhibits Tumor Growth and Metastasis

**DOI:** 10.3390/ijms26104588

**Published:** 2025-05-10

**Authors:** Aliyah L. Bocachica-Adorno, Adriana Y. Aponte-Ramos, Paola S. Rivera-Fuentes, Natalia P. Espinosa-Ponce, Luz V. Arroyo-Cruz, Taotao Ling, Naydi Pérez-Ríos, Sona Rivas-Tumanyan, Israel A. Almodóvar-Rivera, Carlos Barreto-Gamarra, Maribella Domenech-García, Fatima Rivas, Michelle M. Martínez-Montemayor

**Affiliations:** 1 Department of Biology, University of Puerto Rico at Bayamón, Bayamón, PR 00959, USA; aliyah.bocachica@upr.edu (A.L.B.-A.); natalia.espinosa@upr.edu (N.P.E.-P.); 2 Department of Biology, Inter American University of Puerto Rico at Bayamón, Bayamón, PR 00957, USA; yanellyaponte6@gmail.com; 3 Department of Biology, University of Puerto Rico at Río Piedras, San Juan, PR 00935, USA; paola.rivera115@upr.edu; 4 Department of Biochemistry, Universidad Central del Caribe-School of Medicine, Bayamón, PR 00960, USA; luz.arroyo@uccaribe.edu; 5 Department of Chemistry, Louisiana State University, Baton Rouge, LA 70803, USA; tling@lsu.edu (T.L.); frivas@lsu.edu (F.R.); 6 Hispanic Alliance for Clinical and Translational Research, University of Puerto Rico-Medical Sciences Campus, San Juan, PR 00936, USA; naydi.perez@upr.edu (N.P.-R.); sona.tumanyan@upr.edu (S.R.-T.); 7 Department of Surgical Sciences and the Office of the Assistant Dean for Research, School of Dental Medicine, University of Puerto Rico, San Juan, PR 00936, USA; 8 Department of Mathematical Sciences, University of Puerto Rico at Mayagüez, Mayagüez, PR 00689, USA; israel.almodovar@upr.edu; 9 Department of Chemical Engineering, University of Puerto Rico at Mayagüez, Mayagüez, PR 00689, USA; carlos.barreto7@upr.edu (C.B.-G.); maribella.domenech@upr.edu (M.D.-G.)

**Keywords:** ergosterol peroxide, breast cancer, mitochondria

## Abstract

Ergosterol peroxide (EP) triggers apoptosis pathways by inducing reactive oxygen species (ROS) in TNBC cell lines. Excess ROS production is associated with major damage to mitochondria. We hypothesized that EP may act through ROS-induced mitochondrial dysfunction. Therefore, we performed a series of assays that assessed mitochondrial membrane potential (MMP), cellular respiration, and glycolysis in TNBC models. Cardiomyocytes derived from human-induced pluripotent stem cells were chosen as a non-cancerous model because of their high mitochondrial content. Two in vivo TNBC models were used to quantify the effect of EP on tumor volume and metastases. EP reduced MMP and disrupted mitochondrial functions exclusively in TNBC cells. In vivo EP was effective in reducing tumor volume without affecting liver function. There was also a significant decrease in metastasis to the lung, liver, and cancer stem cells following treatment. These results suggest EP is a promising therapy for TNBC.

## 1. Introduction

The most prevalent cancer among women in the United States is breast cancer (BC), comprising 32% of all newly reported annual cases [1]. Globally, BC is the primary cause of death from malignant tumors in women [2]. Triple-negative breast cancer (TNBC), an aggressive BC subtype, accounts for 15–20% of all BC diagnoses and is the most significant cause of mortality in women with BC [3].

TNBC tumors are characterized by a lack of expression of the estrogen receptor (ER), progesterone receptor (PR), and the human epidermal growth factor receptor (HER2). While there is a variety of therapies for these BC subtypes, systemic chemotherapy remains the frontline option for TNBC [4]. TNBC tumors are highly invasive and often metastasize to visceral organs (usually the lungs, liver, brain, and bones). The average survival time if TNBC metastasizes is <18 months [3].

Systemic chemotherapy does not discriminate between cancer cells and healthy cells. This approach is associated with poor patient response, toxicity, and drug resistance [5]. There is a strong need for novel, less toxic therapeutics that elicit a more robust response for women battling this aggressive form of BC. Agents derived from natural sources, such as fungi or plants, provide lead compounds with anti-cancer potential. These natural agents are cost-effective and have minimal side effects. Natural products such as cordycepin, ailanthone, and *Ganoderma lucidum* have potential as natural treatments for TNBC [6].

We previously reported the pro-apoptotic and anti-tumor effects of *Ganoderma lucidum*, a medicinal mushroom, in TNBC models [7]. Further research identified ergosterol peroxide (EP), a compound derived from *G. lucidum*, as a potent and selective anti-cancer agent. When EP enters TNBC cells, it generates reactive oxygen species (ROS). Cancer cells have higher ROS levels than normal cells but maintain redox balance. Therapies that induce ROS disrupt this balance by increasing ROS levels and/or inhibiting antioxidant processes. Endoperoxide compounds like EP that induce ROS production have multiple advantages over chemotherapy; they are selective, have multiple mechanisms of action, and have the potential for enhanced delivery systems.

We and others have shown that EP-induced ROS generation and subsequent apoptosis are attenuated by ROS-generating enzyme inhibitors and antioxidants like N-acetylcysteine, indicating that ROS plays a crucial role in EP-mediated apoptotic cell death [8]. The selectivity may be due to the differing mitochondrial membrane potential (MMP) in cancer cells vs. non-cancerous cells [8,9,10]. Mitochondria are a fundamental part of tumor growth and progression, determining the aberrant energy metabolism of malignant cells and regulating cell death by apoptosis and necrosis [11]. Here, we build on our previous findings by investigating the effects of EP on mitochondria, including the selective activity, potential toxicity, and underlying mechanisms of action. Furthermore, we provide evidence of EP efficacy in tumor volume reduction and metastasis in TNBC animal models.

## 2. Results and Discussion

### 2.1. EP Affects Mitochondrial Membrane Potential in TNBC Cells

Excess ROS directly damages mitochondrial DNA (mtDNA), impairs the electron transport chain, and causes membrane destabilization and Ca^2+^ dysregulation, weakening antioxidant defenses. Together, these processes trigger the cycle of oxidative stress, leading to mitochondrial dysfunction and cell death [7]. In a previous study, we reported that EP selectively triggers apoptosis pathways in TNBC cell lines via the production of ROS [9]. Here, we assess whether this selective effect is due to EP’s effect on MMP using tetramethylrhodamine methyl ester (TMRM) and JC-1 assays. TMRM and JC-1 are widely accepted assays that measure mitochondrial outer membrane permeabilization (MOMP) [10]. These methods are commonly used as reliable proxies for MMP due to the well-documented loss of MMP that follows MOMP activation. TMRM is an intensity-based, cell-permeant dye that accumulates in active mitochondria with intact membrane potential. A decrease in TMRM fluorescence denotes mitochondrial depolarization, indicating a cell is under stress or in the process of apoptosis [11]. JC-1 is a lipophilic cationic dye that forms J-aggregates in healthy cells, indicated by intense red fluorescence, and J-monomers in cells with reduced MMP, indicated by green fluorescence. EP dose was determined as previously described [8].

**Figure 1:**
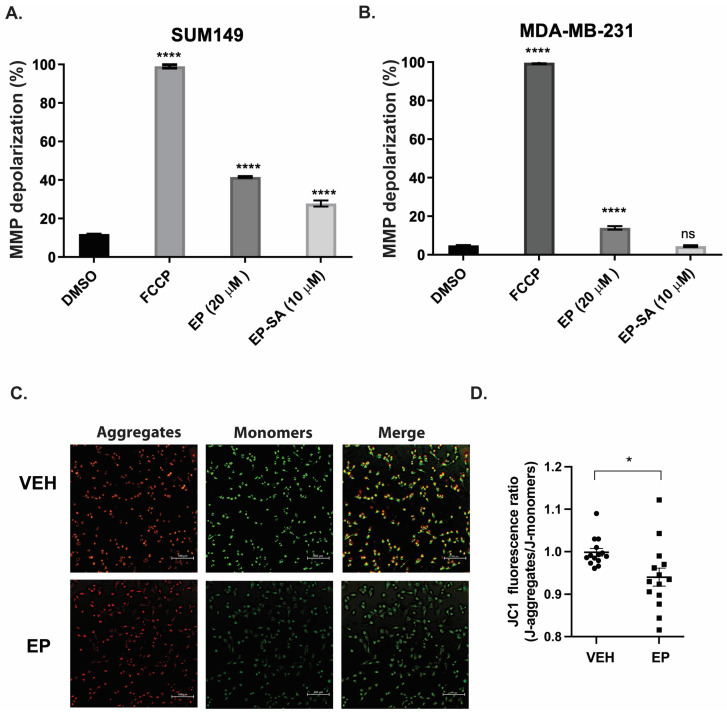
**Ergosterol peroxide (EP) disrupts the mitochondrial membrane potential (MMP) in TNBC cells:** (**A**) SUM-149 or (**B**) MDA-MB-231 cells treated with either 0.2% DMSO (Veh, control), 20 µM EP, 10 µM EP-SA, or 20 µM FCCP for 24 h. MMP was assessed using TMRM fluorescent dye. EP significantly induced MMP depolarization compared to the vehicle. Bars depict the mean ± SEM of three independent experiments. **** *p* < 0.0001, ns: not significant. (**C**) MMP assessment using the JC-1 assay. Red depicts J-aggregates that accumulate in healthy cells. Green represents J-monomers that accumulate in unhealthy/apoptotic cells. (**D**) J-Aggregates and J-monomers were individually counted, and data presented as a ratio of J-aggregates to J-monomers. Images were taken at 20×. Results show the mean ± SEM of three independent experiments. * *p* ≤ 0.05.

EP significantly reduced MMP potential in TNBC cell lines (SUM-149 and MDA-MB-231) compared to controls (Figure 1A,B). The positive control trifluoromethoxy carbonylcyanide phenylhydrazone (FCCP) decoupled the mitochondria, resulting in MMP collapse in around 100% of the TNBC cell lines. We also tested the effect of an EP derivative, EP-SA [9], which only produced a significant effect in SUM-149 cells (Figure 1A). The reduction in MMP in MDA-MB-231 cells treated with EP was confirmed with the JC-1 assay. EP treatment significantly (*p* < 0.05) decreased the J-aggregates to the J-monomers ratio in these cells (Figure 1C,D). This ratio is directly correlated to the polarization of the mitochondrial membrane, indicating a disruption in MMP following the administration of EP. Together, these results indicate that EP treatment depolarizes the mitochondrial membrane, which is a key feature of the selective properties of EP.

### 2.2. EP Selectively Reduces Cellular Respiration and Glycolysis in TNBC Cells

**Figure 2:**
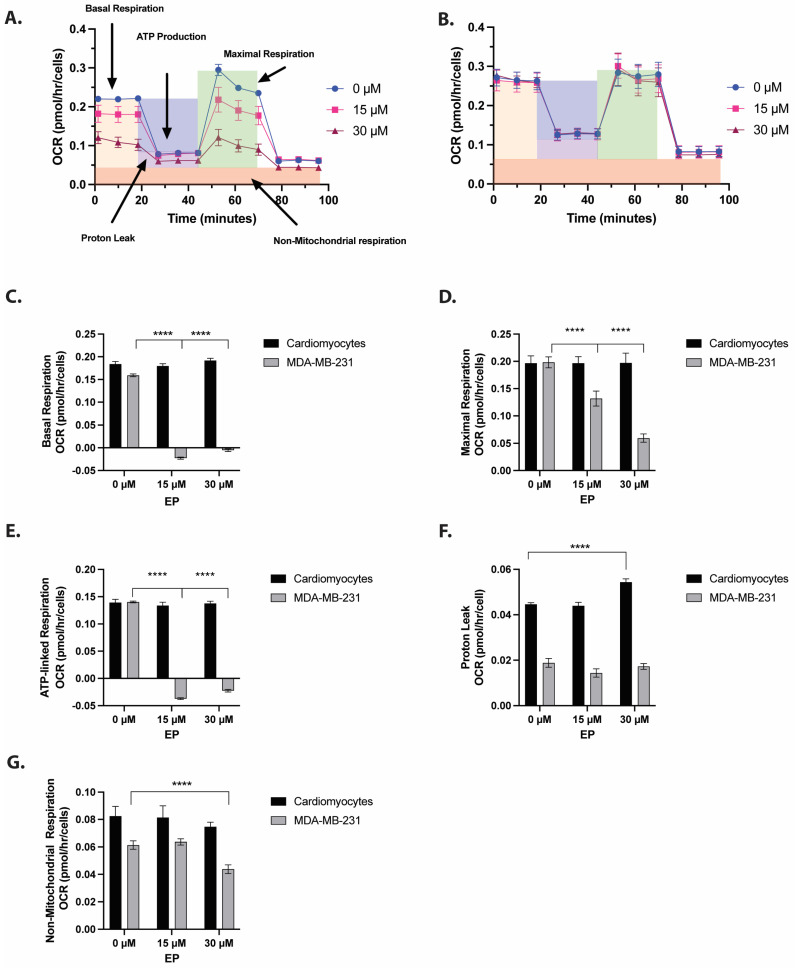
**Ergosterol peroxide (EP) decreases cellular respiration in TNBC cells:** Cardiomyocytes differentiated from human-induced pluripotent stem cells and MDA-MB-231 TNBC cells were treated with 0–30 μM EP for 24 h. (**A**) OCR was significantly decreased in TNBC cells starting at 15 μM particularly ATP production and maximal respiration. (**B**) The mitochondrial stress test revealed that oligomycin further decreased OCR only in TNBC cells. (**C**) Basal respiration was significantly reduced in TNBC cells starting at 15 μM. (**D**) Maximal respiration was significantly reduced starting at 15 μM. (**E**) ATP-linked respiration was significantly reduced starting at 15 μM. (**F**) Proton leak was not affected in cancer cells and was slightly increased in cardiomyocytes at 30 μM. (**G**) Non-mitochondrial respiration was significantly reduced at 30 μM. Cardiomyocytes showed no significant changes at any EP concentration. The mitochondrial stress test confirmed that EP selectively impaired mitochondrial function in TNBC cells. A two-way ANOVA with Bonferroni’s multiple comparison test was performed. Bars show the mean ± SEM of three independent experiments. **** *p* < 0.0001.

Mitochondrial metabolism has emerged as a viable target for cancer therapy. The anti-tumor effects of agents that inhibit mitochondrial bioenergetic capacity stem from disruptions in the electron transport chain [8]. To investigate the effects of EP on mitochondrial metabolism, we measured the oxygen consumption rate (OCR) of TNBC cells. Cardiomyocytes, differentiated from human pluripotent stem cells, were used as a non-cancerous control. These cells were specifically chosen as controls for their high mitochondrial content [12] (Figure 2A,B). We found that the basal OCR in MDA-MB-231 TNBC cells decreased significantly (*p* < 0.0001) in response to the 15 µM and 30 µM EP doses (Figure 2C). A mitochondrial stress test using oligomycin (mitochondrial complex V inhibitor) confirmed that this effect was limited to TNBC cells (Figure 2D). ATP production and maximum respiration significantly decreased in EP-treated cells (Figure 2E). Proton leak was not affected in the cancer cells and was slightly increased at 30 µM EP in cardiomyocytes (Figure 2F). It is possible that at this higher concentration (IC_50_ = 20 mM in 72 h) and shorter duration, EP does not kill the cardiomyocytes but instead causes a non-lethal increase in proton leak.

EP also impacted non-mitochondrial respiration in TNBC cells (Figure 2G). These results confirm that non-lethal concentrations of EP disrupt mitochondrial function in treated cells [9,13,14]. EP had no adverse effects on cardiomyocytes at 15 µM, and a slight increase in proton leakage at 30 µM concentration (Figure 2F), with the remaining parameters intact. Our previous studies document non-toxic effects of EP in MCF10A, HMEC and BJ cells [9,13,14]. This highlights EP’s potential selectivity for cancer cells and addresses a significant limitation in cancer therapeutics. The observed decrease in basal OCR, ATP production, and maximal respiration verify that EP disrupts the mitochondrial function in TNBC cells by impairing the electron transport chain and diminishing the bioenergetic capacity.

**Figure 3:**
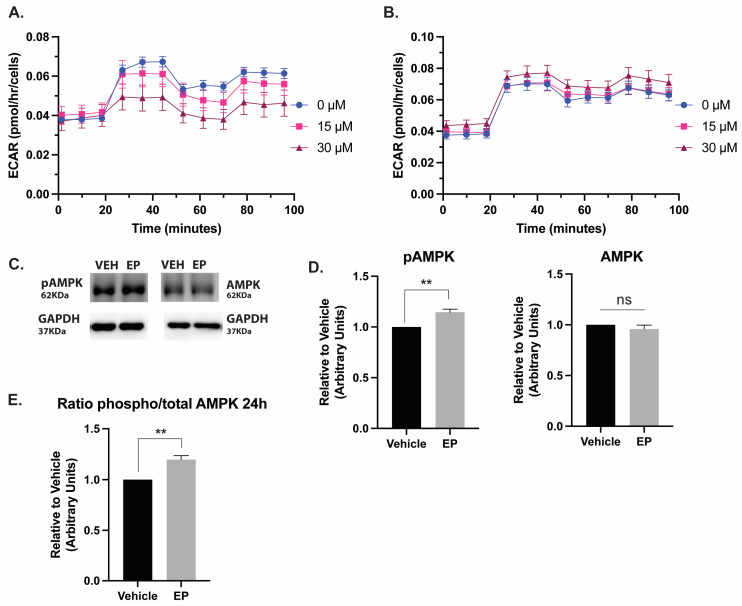
**EP decreases glycolysis in TNBC cells:** (**A**) MDA-MB-231 TNBC cells or (**B**) cardiomyocytes differentiated from human-induced pluripotent stem cells were treated with 0–30 µM EP for 24 h. ECAR was affected by EP at 30 μM only in cancer cells. A two-way ANOVA with Bonferroni’s multiple comparison test was performed. (**C**) Immunoblots of MDA-MB-231 cells treated with vehicle or 20 µM EP for 24 h. Tumor lysates were probed for the indicated proteins. (**D**,**E**) Densitometry quantification analysis of total and phospho-proteins; the intensities were normalized to its loading control and plotted. A two-tailed unpaired t-test was performed; ** *p* < 0.01, ns: not significant. Results are shown as Mean ± SEM of three independent experiments.

Next, we assessed glycolysis by measuring the extracellular acidification rates (ECAR) in our cancerous (MDA-MB-231 TNBC cells) and non-cancerous (cardiomyocyte) models. Cells were treated with 15 µM or 30 µM of EP and 2-deoxyglucose (2-DG), which is a glucose substitute that is not metabolized by cells. We found EP significantly decreased glycolysis at the 30 µM dose in TNBC cells (Figure 3A,B). We also identified a significant increase in p-AMPK activation (Figure 3C–E), suggesting that TNBC cells were attempting to activate glycolytic pathways to maintain ATP production and compensate for impaired mitochondrial function. Finally, we report that non-lethal doses of EP (5 μM) cause DNA damage to TNBC cells after 72 h of treatment (Figure S1).

Our results confirm that EP selectively reduces cellular respiration and glycolysis in TNBC cells (Figure 2 and Figure 3). Previous studies have shown that TNBC cells rely heavily on mitochondrial oxidative phosphorylation (OXPHOS) for energy production and survival. TNBC cells have high glucose uptake, increased lactate production, and reduced mitochondrial respiration, which makes them dependent on glycolysis to generate ATP [15]. AMPK is a master regulator of cell metabolism. When there is low cell energy charge, AMPK is activated by the phosphorylation of threonine 172 due to a high AMP/ATP ratio [16]. AMPK activation has also been associated with apoptosis regulation following exposure to metabolic stressors [17]. γH2AX becomes phosphorylated on serine 139 as a reaction to double-strand breaks, where it is primarily responsible for the recruitment of repair complexes at the specific site of the DNA damage [18]. When AMPK is activated, cells that have inappropriate metabolic resources to repair DNA damage become more apoptotic and display cell cycle arrest [16]. Inhibiting glycolysis in these cells with EP has proven effective in inducing cancer cell death. Our results provide insights into the mechanism of EP therapy response and suggest a strategy where a mitochondria-disruptive agent, such as EP, could improve responses for patients suffering from this intractable disease. EP’s ability to disrupt these critical metabolic pathways may underlie its capacity to induce apoptosis in TNBC cells.

### 2.3. EP Reduces Tumor Volume in a Model of TNBC

We evaluated EP’s treatment potential in two in vivo experiments that measured its effect on tumor volume and metastases. Both experiments used female hairless severe combined immunodeficient (SHO-SCID) mice injected with the TNBC cell line MDA-MB-231-GFP in the 4th right mammary fat pad. EP’s potential toxicity was evaluated via hepatic enzyme levels.

**Figure 4:**
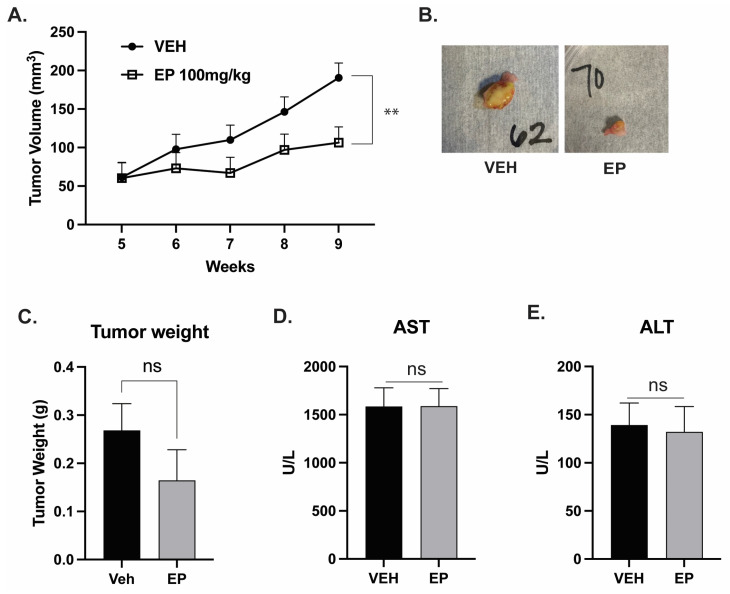
**EP significantly decreases tumor volume without causing toxicity:** Female hairless severe combined immunodeficient (SHO-SCID) mice (n = 14/tx) were injected with MDA-MB-231-GFP (1.0 × 10^6^) cells in their 4th right mammary fat pad. Mice were treated with intraperitoneal injections of either vehicle (2% Tween 80, 2% ethanol, 96% sterile distilled water) or EP (100 mg/kg BW every two to three days). (**A**) Tumor volume was recorded twice a week using caliper measurements and analyzed as described in the Materials and Methods section. (**B**) Tumors were excised on the 9th week post-treatment. EP significantly reduced tumor volume by 44% compared to the vehicle treatment. ** *p* < 0.01 (**C**) Tumor weights were obtained at the end of the study. EP reduces tumor weight by 40%, ns: not significant. Bars show mean ± SEM. Blood was collected to analyze: (**D**) AST and (**E**) ALT, which showed that EP did not affect enzyme levels. Bars show mean ± SEM.

In the first experiment evaluating tumor volume (Figure 4), mice were treated with intraperitoneal injections of either vehicle (2% Tween 80, 2% ethanol) or EP (100 mg/kg body weight (BW)) every three days for 9 weeks. Mice were weighed once a week, and tumor volume was measured twice a week with precision calipers. By week 9, we identified a significant treatment effect (*p* < 0.002), indicating a decrease in tumor volume in mice treated with EP compared to those treated with the vehicle (Figure 4A). Tumors in EP-treated mice were generally smaller, and 25% of these mice had no detectable tumors by the end of the study (Figure 4B). However, the results for tumor weight did not reach significance (Figure 4C). Body weight was not affected by EP treatment [14].

To determine if EP treatment had toxic effects in mice, we measured the levels of hepatic enzymes, aminotransferase (AST), and alanine aminotransferase (ALT). AST and ALT levels (Figure 4D,E) were within normal ranges for both groups, and there was no significant difference between the EP-treated mice and the vehicle-treated mice. These results indicate that following 9 weeks of EP treatment, mice had normal and functional liver health and had no toxic effects associated with EP treatment.

### 2.4. EP Significantly Reduces TNBC Metastasis in the Lungs and the Liver

In the second in vivo experiment, we evaluated the effect of EP on metastases. More than one third of patients with TNBC present with distant organ metastasis, which is most common in the lung and liver [19]. Metastatic TNBC drastically decreases overall patient survival (OS) to ~15 months [20]. We evaluated EP’s potential to decrease the metastatic potential of TNBC in the lung and liver of the female SCID mice. Mice received either a vehicle (10% ethanol, 90% corn oil) or EP at a dose of 100 mg/kg BW, which was administered via a 100 µL oral gavage for 13 weeks. At the end of this period, mice were euthanized, the organs were excised, washed, and observed under a stereoscope. The gallbladder was removed before images were taken.

**Figure 5:**
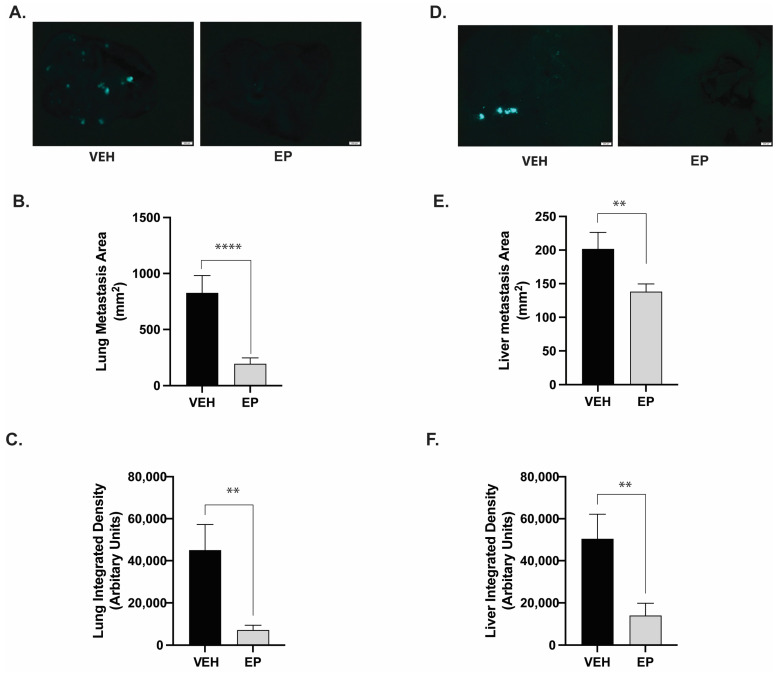
**EP significantly decreases TNBC metastasis in lungs and liver:** Mice (n = 18/tx) were injected with MDA-MB-231-GFP cells and received treatment for 11 weeks. After euthanasia, lungs and livers were excised and analyzed for metastasis using fluorescence microscopy, which was followed by quantitative image analysis. Fluorescent areas indicate the presence of MDA-MB-231-GFP cells. (**A**) Representative images of lungs from mice following either vehicle (90% corn oil and 10% ethanol) or EP (100 mg/kg of BW) treatment. Scale bar = 200 μm. (**B**) Quantitative image analysis demonstrates that EP significantly reduces lung lesion size. **** *p* < 0.0001. (**C**) EP significantly decreases the number of TNBC cells in lung metastatic foci. ** *p* < 0.01. (**D**) Representative images of livers from mice following either vehicle or EP treatment. Scale bar = 200 μm. (**E**) Image analysis indicates EP causes a significant reduction in the liver lesion size. ** *p* < 0.01. (**F**) EP significantly reduces the number of TNBC cells in liver metastatic foci. ** *p* < 0.01. Bars show the mean ± SEM.

Ventral and dorsal images of each organ were used to quantify the metastatic foci. ImageJ was used to quantify the area, which represents the lesion size, and integrated density, which represents the number of cells present in each metastatic foci. EP treatment resulted in a significant (76%) reduction in the lung metastasis area, indicating a reduced lesion size (Figure 5A,B). EP also decreased the number of TNBC cells residing in lung metastatic foci by 84% (represented by integrated density) (Figure 5C) [21]. We found similar results in the liver. EP significantly reduced metastasis to the liver compared to the vehicle group (Figure 5D) with a 30% decrease in the liver lesion size (Figure 5E) and a 72% reduction in the number of metastatic TNBC cells present in the liver metastatic foci (Figure 5F).

The current findings expand our established research program investigating EP as an anti-cancer agent. Beginning with the initial extraction of EP from *G. lucidum* [8], we subsequently developed synthetic routes to generate EP, creating a library of compounds with anti-cancer activity and favorable safety profiles [9,10]. We found that EP selectively induced apoptosis in cancer cells by generating ROS [8]. We hypothesized that this selectivity may stem from differences in MMP alteration by EP between TNBC and normal cells. In the current study, we show that EP disrupts MMP and significantly reduces cellular respiration and glycolysis in TNBC models. In vivo, EP treatment resulted in significant reductions in tumor volume and metastases to the lungs and liver. Notably, the TNBC cell line used in this study, MDA-MB-231, contains a highly metastatic population enriched (>90%) in CD44^+^/CD24^−^ cancer stem cells (CSCs) [22]. Obtaining these results in this highly metastatic cell line further underscores EP’s therapeutic potential.

CSCs regulate processes such as angiogenesis, proliferation, survival, invasion, chemoresistance, and metastasis. CSCs also exhibit higher mitochondrial activity and preferentially use respiration and OXPHOS as energy sources. We previously described *G. lucidum*’s effect on CSCs [23]. Here, we show EP’s dose-dependent reduction in TNBC CSCs, via decreased mammosphere formation and stemness marker abundance, as shown in Figure S2. Together, our findings suggest that EP is a promising therapy for TNBC.

## 3. Materials and Methods

### 3.1. Cell Culture and Reagents

Cell lines MDA-MB-231 (ATCC^®^ HTB-26TM) and MCF-10A (ATCC^®^ CRL-10317) were obtained from the American Type Culture Collection (ATCC, Manassas, VA, USA), while SUM-149 cells were obtained from BioIVT (Westbury, NY, USA). MDA-MB-231 were cultured in Dulbecco’s Modified Eagle’s Medium (DMEM) (Life Technologies, Rockville, MD, USA) supplemented with 10% fetal bovine serum (FBS) (Corning, Corning, NY, USA), while SUM-149 were cultured in Ham’s F12 (Life Technologies, Rockville, MD, USA) with 10% FBS. The human noncancerous mammary epithelial cell line MCF-10A was cultured in DMEM/Ham’s F12 (Life Technologies, Rockville, MD, USA) with 10% horse serum (Sigma Aldrich, St. Louis, MO, USA). All cell lines were incubated at 37 °C and were maintained in an atmosphere containing 5% CO_2_. Cells were tested regularly to ensure they were free from mycoplasma infection using the Mycoplasma Detection Kit (ASB-1310001, Nordic BioSite AB, Täby, Sweden). MDA-MB-231-GFP cells (a kind gift of Dr. Suranganie Dharmawardhane, University of Puerto Rico Medical Sciences Campus) were cultured as previously described [24]. EP, the main reagent utilized, was synthesized as described [9,13,14].

### 3.2. JC-1 Mitochondrial Membrane Potential Assay

MDA-MB-231 cells (1.5 × 10^5^ cells/mL/well) were seeded in 6-well tissue culture plates (92006, TPP, Trasadingen, Switzerland) and cultured for 24 h at 37 °C, 5% CO_2_. Cells were then treated with either vehicle (0.2% DMSO) or 20 µM EP for 24 h. After the treatment period, cells were stained with the JC-1 Staining Solution (JC-1 Mitochondrial Membrane Potential Assay Kit No. 10009172, Cayman Chemical Company, Ann Arbor, MI, USA) following the manufacturer’s protocol. Briefly, the staining solution was directly dispensed in cell culture medium (100 µL/mL of medium) and incubated for 20 min. Visualization was carried out with the Eclipse Ts2 Inverted Routine Microscope (Nikon, Melville, NY, USA). Images (n = 5 images/well) were obtained using two filters: TRITC (Red) and FITC (Green) at a 20× magnification. This was followed by the manual tabulation of red J-aggregates and green J-monomers to establish a J-aggregates to J-monomers ratio in each treatment.

### 3.3. Tetramethylrhodamine Methyl Ester Perchlorate (TMRM) Assay

The TMRM assay was performed as described [24]. Briefly, SUM-149 cells (5 × 10^5^ cells/well) or MDA-MB-231 cells (2 × 10^5^ cells/well) were seeded in 6-well plates for 24 h at 37 °C, 5% CO_2_. Cells were then treated with either vehicle (DMSO 0.2%), 20 µM EP, or 20 μM FCCP (24 h). Processing was carried out using the Image-iT TMRM Reagent (I34361, Invitrogen, Carlsbad, CA, USA). FCCP was used as the positive control and was added for 10 min prior to TMRM staining. The cell culture medium was removed, and cells were washed with phosphate-buffered saline (PBS), trypsinized, or resuspended in PBS supplemented with 1% FBS and analyzed by flow cytometry.

### 3.4. Seahorse XF Mito Stress Test

Cardiac cells were derived from the human-induced pluripotent stem cells (hiPSCs) WTC11 cell line (GM25256, Coriell Institute, Camden, NJ, USA) and were cultured and differentiated according to the GiWi protocol [25], as described in [12]. The number of cardiomyocytes was measured by flow cytometry and estimated based on the fraction of cardiac troponin T (cTNT)-positive cells. Only cell differentiations with cTNT >70% and cardiac troponin I (cTNI) >30% were considered for this study (Figure S3). For metabolic analysis, cardiomyocytes (8.0 × 10^4^ cells/well) and MDA-MB-231 (2.0 × 10^4^ cells/well) were seeded into XF 24-well cell culture microplates (100777-004, Agilent Technologies, Santa Clara, CA, USA) with a surface area of 0.275 cm^2^ per well. Before seeding, differentiated cardiac cells cultured in 24-well plates (3526, Corning, Corning, NY, USA) were washed with 1× DPBS (P5368, Sigma Aldrich, St. Louis, MO, USA) and enzymatically detached using Accutase (AT-104, Innovative Cell Technologies, San Diego, CA, USA) for 12 min at 37 °C under 5% CO_2_. The cell suspension was neutralized in DMEM/F12 (11320-033, Gibco, Waltham, MA, USA) at a 1:4 ratio, which was followed by centrifugation at 220× *g* for 5 min. Cells were resuspended in RPMI 1640 medium supplemented with B-27 Plus (17504–044, Gibco, Waltham, MA, USA) and transferred to Matrigel-coated XF 24-well microplates (growth factor reduced, CB-40230A, Fisher Scientific, Waltham, MA, USA). After 24 h of incubation at 37 °C, the culture medium was replaced with Seahorse assay medium composed of DMEM (103575–100, Agilent, Santa Clara, CA, USA) supplemented with 2 mM L-glutamine (103579–100, Agilent, Santa Clara, CA, USA), 1 mM pyruvate (103578–100, Agilent, Santa Clara, CA, USA), and 10 mM glucose (103577–100, Agilent, Santa Clara, CA, USA). Cells were pre-equilibrated in this medium for 1 h at 37 °C in a non-CO_2_ incubator. Following this, the Seahorse sensor cartridge was hydrated and loaded with mitochondrial stress test compounds: oligomycin (1 µM, Port A), FCCP (0.5 µM, Port B), and a rotenone/antimycin A mix (0.5 µM, Port C). At the end of the assay, cells were fixed with 4% paraformaldehyde (15710, Electron Microscopy Sciences, Hatfield, PA, USA) for 20 min and washed with 1× DPBS. Nuclei were stained using Hoechst 33342 (H3570, Invitrogen, Carlsbad, CA, USA; 1:1000 dilution in 1× PBS). After staining, cells were washed three times with 1× PBS. Entire wells were captured at 2× magnification using the Keyence BZ-X810 microscope (Keyence Corporation, Itasca, IL, USA), and nuclear counts were obtained using the “Find Maxima” function in ImageJ software (Version 1.53a, NIH, Bethesda, MD, USA). Metabolic values were normalized to total nuclei counts per well.

### 3.5. Immunoblots

MDA-MB-231 TNBC cells were cultured to 80% confluency and treated with either vehicle (0.2% DMSO) or EP at 20 µM for 24 or 72 h. Following treatment, cells were washed with PBS and lysed in NP-40 lysis buffer containing protease inhibitors leupeptin (L2884), pepstatin (P5318), and chymostatin (C7268) (Sigma-Aldrich, St. Louis, MO, USA). Lysates were vortexed intermittently on ice for 10 min and centrifuged at 14,000 RPM for 10 min at 4 °C. Total protein was quantified using the Precision Red protein assay kit (ADV02-A), Cytoskeleton, Inc. Denver, CO, USA). Equal amounts of protein (30 µg) were resolved on 10% SDS-PAGE gels and transferred to PVDF membranes (IPVH00010, Immobilon^®^-P, Millipore Sigma, Burlington, MA, USA). Membranes were blocked with 5% BSA in TBST and incubated overnight at 4 °C with primary antibodies from Cell Signaling Technology (Danvers, MA, USA) at the dilutions recommended by the manufacturer. The following antibodies were used: AMPKα (23A3) (#2603P), Phospho-AMPKα (Thr172) (40H9) (#2535P), SOX2 (D6D9) (#3579S), NANOG (D73G4) (#4903S), OCT4 (C30A3) (#2840S), and GAPDH (#5174S) as a loading control. Membranes were then incubated with HRP-conjugated anti-rabbit IgG (A9169, 1:10,000) or anti-mouse IgG (A9044, 1:20,000) secondary antibodies (Sigma-Aldrich, St. Louis, MO, USA) for 1 h at room temperature (RT). Protein bands were visualized using the SuperSignal™ detection system (34096, Thermo Fisher Scientific, Waltham, MA, USA) and imaged using the BioSpectrum Imaging System (UVP LLC, Upland, CA, USA). The integrated density of the bands of interest was quantified using ImageJ software (NIH, Bethesda, MD, USA). The quantification of proteins was ensured by normalizing the integrated densities of each band of interest for the antibody to the integrated density of the same immunoblotted lysate for GAPDH as described by us [9,26,27,28,29]. Arbitrary units are equal to the normalized integrated density of each protein relative to the vehicle control.

### 3.6. Immunocytochemistry

The fixing, permeabilization, and blocking solutions were prepared in 1× PBS. 1 × 10^5^ SUM-149 TNBC cells were seeded in coverslips and treated with vehicle, 5 μM EP, or 1 μM Doxorubicin for 72 h. After the incubation, cells were fixed with 4% paraformaldehyde for 15 min, washed with 1× PBS, and permeabilized with 0.5% Triton X-100 for 10 min at room temperature. Cells were washed with 1× PBS and blocked with 5% BSA for 1 h at room temperature. For labeling, fixed cells were incubated 2 h with a primary antibody against γ-H2AX (20E3,1:200, Cell Signaling, Danvers, MA, USA) and washed. Coverslips were incubated with anti-Rabbit Alexa 488 (1:750, #4412S, Cell Signaling, Danvers, MA, USA) for 1 h at room temperature. After three washes with 1X PBS, cells were incubated for 1 min at room temperature with 1 μg/mL of DAPI (D9542, Sigma Aldrich, St. Louis, MO, USA) and washed. Cells were mounted on slides with antifade medium (Life Technologies, Rockville, MD, USA). Micrographs were taken using an Olympus inverted microscope (Olympus, Center Valley, PA, USA). Cells containing > 5 γ-H2AX distinct foci were quantified as described by us [30].

### 3.7. In Vivo Study

Female SHO-SCID mice (3-week-old, Charles River Laboratories, Inc., Wilmington, MA, USA) were maintained under pathogen-free conditions in Hepa-filtered cages under controlled light (12 h light and dark cycle), temperature, and humidity as in [14,21,22,28,29,30,31,32]. Throughout the experiment, the animals were fed an autoclaved AIN 76-A phytoestrogen-free diet (Tek Global, Harlan Teklad, Madison, WI, USA) and water ad libitum.

#### 3.7.1. Tumor Model

MDA-MB-231-GFP (1 × 10^6^ cells) in starving media: reduced growth factor Matrigel (BD Biosciences, San Jose, CA, USA) (1:1) were injected into the fourth right mammary fat pad of female nude mice under isoflurane inhalation as described in [14,21,23,28,29,30,31,32]. After tumor establishment (1 week post-inoculation), the animals were randomly assigned to experimental treatment groups. About 1–2 animals per group were eliminated due to failure of tumor take, small or too large tumor area in 1 week, or penetration of the peritoneum that resulted in immediate GFP fluorescence in the intestines. Mice with similar tumor areas quantified by the integrated density of fluorescence images were selected for further study.

#### 3.7.2. Treatment Administration

*Study 1.* Female SHO-SCID mice (n = 14/group) divided in two groups (vehicle and EP) were injected intraperitoneally (i.p.) with either vehicle (2% Tween 80, 2% ethanol, 96% sterile distilled water) or EP (100 mg/kg BW) in a 100 µL volume three times per week. Treatments continued until euthanized at week 9. *Study 2*. Female SHO-SCID mice (n = 18/group), divided into two groups (vehicle and EP), were orally gavaged with either vehicle (90% corn oil, 10% ethanol) or EP (100 mg/kg BW) in a 100 µL volume once a day. Treatments continued until euthanization at week 13.

#### 3.7.3. Analysis of Metastases

Following euthanasia, the lungs and the liver were excised and stored in liquid N_2_. Stored organs were thawed, and the gallbladder in the liver was removed. Organs were cleaned with 1× PBS before imaging and analysis using an Olympus MV10 fluorescence macro zoom stereoscope (Olympus, Center Valley, PA, USA) and Olympus DP71 microscope digital camera (Olympus, Center Valley, PA, USA) as in [21]. Each organ was imaged in the ventral and dorsal orientations. Fluorescent lesions (green component of RGB images), area (size of the lesions), and integrated density (number of metastatic foci) were quantified using ImageJ software (National Institutes of Health, Bethesda, MD, USA). To eliminate false positives, areas identified as metastases were also validated by visual inspection.

### 3.8. Statistical Analyses

In vitro data was exported to the Graph Pad Prism v. 10.2.3 program, and statistical analyses were carried out via a two-tailed unpaired t-test or ANOVA. *p* values were determined using Student’s t-test, and values ≤ 0.05 were considered significant.

*Animal study 1:* To evaluate the effect of EP treatment on tumor volume in an experimental TNBC model in mice, baseline tumor measurements (e.g., area volume) were described using measurements of central tendency and dispersion (such as mean, standard deviation, median, and interquartile range) and compared between treatment groups (EP, control) using a *t*-test. Measurements were averaged in the first five weeks of the study and were used as baseline. To determine the effect of treatment group and time on the remaining weeks (week 1–5 as baseline until week 9) for the average tumor volume as the outcome, linear mixed regression models were employed. Interactions between treatment groups and follow-up time were assessed using the likelihood ratio (LR) test. Regression coefficients and their 95% confidence intervals for the fixed, random and interaction effects were reported. All statistical analyses were performed using STATA, v.18 (StataCorp. 2023. Stata Statistical Software: Release 18. College Station, TX, USA: StataCorp LLC), and *p*-values less than 0.05 were considered to have statistical significance (*p* ≤ 0.05).

*Animal study 2:* Descriptive statistics were computed for the area (size of the lesion) and integrated densities (number of cells) in lung and liver metastatic foci for vehicle and EP. To determine the difference between the vehicle and EP in each metastatic foci, a Poisson regression model was performed using a log link function. For both models, EP was considered to be the reference group. We reported the regression coefficients and the incidence rate ratio (IRR), which allowed us to obtain the expected area or integrated density of lung and liver metastatic foci changes compared to EP as well as their corresponding confidence interval. Statistical significance was performed at the 5% level (*p* < 0.05). All analyses were conducted using the statistical software R 4.3.1.

## 4. Conclusions

Our studies demonstrate that EP is a highly selective and promising anti-cancer agent. EP effectively targets two key vulnerabilities of TNBC cells, their distinct metabolic profile and tumorigenic potential, while exhibiting minimal toxicity to healthy cells. These findings position EP compounds as strong novel therapeutic candidates for this intractable disease.

## Data Availability

The original contributions presented in this study are included in the article. Further inquiries can be directed to the corresponding author.

## Supplementary Materials

The supporting information can be downloaded at https://www.mdpi.com/article/10.3390/ijms26104588/s1.

## Abbreviations

The following abbreviations are used in this manuscript:

EP	ergosterol peroxide
EP-SA	ergosterol peroxide sulfonamide
BC	breast cancer
TNBC	triple-negative breast cancer
ROS	reactive oxygen species
MMP	mitochondrial membrane potential
ER	estrogen receptor
PR	progesterone receptor
HER2	human epidermal growth factor receptor
TMRM	tetramethylrodhamine methyl ester
mtDNA	mitochondrial DNA
FCCP	trifluoromethoxy carbonylcyanide phenylhydrazone
OCR	oxygen consumption rate
ECAR	extracellular acidification rates
2-DG	2-deoxyglucose
OXPHOS	oxidative phosphorylation
SHO-SCID	hairless outbred severe combined immunodeficient
BW	body weight
AST	aspartate aminotransferase
ALT	alanine aminotransferase
Tx	treatment
GLE	*Ganoderma lucidum* extract
CSCs	cancer stem cells
ALDH1	aldehyde dehydrogenase 1
DMEM	Dulbecco’s Modified Eagle’s Medium
FBS	fetal bovine serum
PBS	phosphate-buffered saline
hiPSCs	human-induced pluripotent stem cells
cTNT	cardiac troponin T
cTNI	cardiac troponin I
BSA	bovine serum albumin
RT	room temperature
LR	likelihood ratio
NIGMS	National Institutes of Health-National Institute of General Medical Sciences
NSF	National Science Foundation
CIAS	Common Instrumentation Area & Services
OADRGS	Office of the Associate Dean for Research and Graduate Studies
STCE	Science and Technology Competency Enhancement
INBRE	IDeA Networks of Biomedical Research Excellence
