# Supplementary material for: Ergosterol Peroxide Disrupts Triple-Negative Breast Cancer Mitochondrial Function and Inhibits Tumor Growth and Metastasis
Source: Int J Mol Sci. 2025 May 10;26(10):4588. doi: 10.3390/ijms26104588 (PMC12111164; doi:10.3390/ijms26104588)
Supplement: Supplementary file 1 [file ijms-26-04588-s001.zip › ijms-3602915-supplementary.pdf]

Article

Ergosterol peroxide disrupts triple negative breast cancer mitochondrial function and inhibits tumor growth and metastasis

Aliyah L. Bocachica-Adorno [1], Adriana Y. Aponte-Ramos [2], Paola S. Rivera-Fuentes [3], Natalia P. Espinosa-Ponce [1], Luz V. Arroyo-Cruz [4], Taotao Ling [5], Naydi Pérez-Ríos [6], Sona Rivas-Tumanyan [6,7], Israel Almodóvar-Rivera [8], Carlos Barreto Gamarra [9], Maribella Domenech-García [9], Fatima Rivas [5], and Michelle M. Martínez-Montemayor [4] *

1. Department of Biology, University of Puerto Rico at Bayamón, Bayamón, PR 00959, USA;aliyah.bocachica3@gmail.com (A.B.A.); natalia.espinosa@upr.edu (N.P.E.P.)
2. Department of Biology, Inter American University of Puerto Rico at Bayamón, Bayamón, PR 00957, USA yanellyaponte6@gmail.com (A.Y.A.R.)
3. Department of Biology, University of Puerto Rico at Río Piedras, San Juan, PR 00935, USA; paola.rivera115@upr.edu (P.S.R.F.)
4. Department of Biochemistry, Universidad Central del Caribe-School of Medicine, Bayamón, PR 00960, USA; luz.arroyo@uccaribe.edu (L.V.A.C.)
5. Department of Chemistry, Louisiana State University, Baton Rouge, LA 70803, USA; tling@lsu.edu (T.L.); frivas@lsu.edu (F.R.);
6. Hispanic Alliance for Clinical and Translational Research, University of Puerto Rico-Medical Sciences Campus, San Juan, PR 00936, USA; naydi.perez@upr.edu (N.P.R)
7. Department of Surgical Sciences and the Office of the Assistant Dean for Research, School of Dental Medicine, University of Puerto Rico, San Juan, PR 00936, USA; sona.tumanyan@upr.edu (S.R.T)
8. Department of Mathematical Sciences, University of Puerto Rico at Mayagüez, Mayagüez, PR 00689, USA; israel.almodovar@upr.edu (I.A.A.R.)
9. Department of Chemical Engineering, University of Puerto Rico at Mayagüez, Mayagüez, PR 00689, USA; carlos.barreto7@upr.edu (C.B.G.); maribella.domenech@upr.edu (M.D.G)

* Correspondence: michelle.martinez@uccaribe.edu or mmmtz92@gmail.com; Tel.: +1-787-798-3001 (ext. 2152)

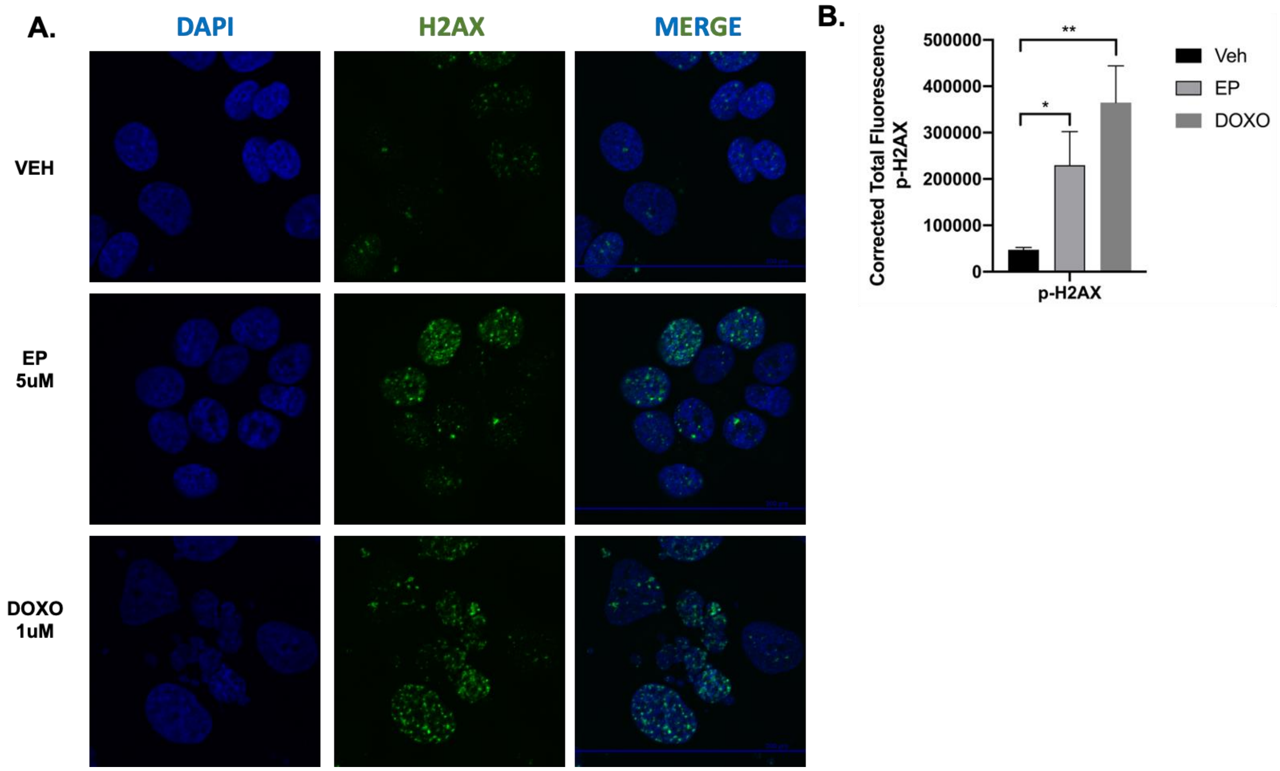

Figure S1. *EP induces DNA Damage in TNBC cells.* ***(A)*** *SUM-149 TNBC cells were treated with Vehicle, EP (5µM) or positive control Doxorubicin (Doxo, 1µM) for 72h. Cells were immunoassayed for anti-p-H2AX (green), stained with DAPI (blue). EP and Doxo induce DNA damage as shown by increased nuclear foci. (****B****).* *Quantification of p-H2AX fluorescence confirms a significant increase in EP (P<0.05) treated cells. Bars represent mean ± SEM of n=3 independent experiments. *P<0.05, **P<0.01. Blue scale bar = 200 µm*

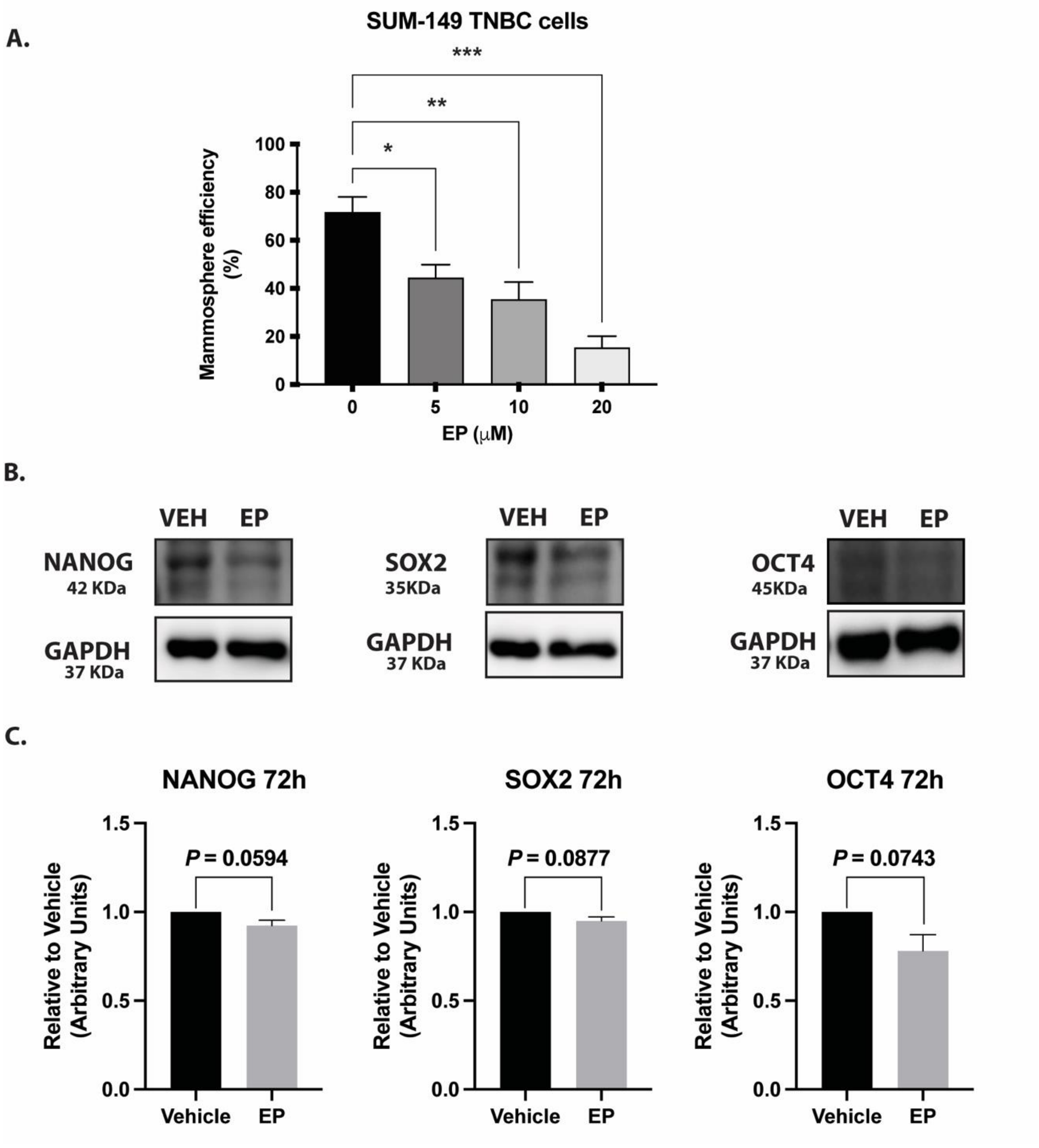

Figure S2. *EP decreases TNBC stem cells. (**A**) SUM-149 TNBC (1.0 x 10^4) cells were seeded for three generations to form mammospheres in non-serum non-adherent culture conditions in the absence (Vehicle) or presence of EP for 72h. Our results show a dose dependent decrease in the percent of formation of the mammospheres. Bars represent mean ± SEM of n=3 independent experiments. *P<0.05, **P<0.01, ***P<0.001. MDA-MB-231 TNBC cells were treated with Vehicle or EP for 72h. (**B**) Total protein was extracted from treated cells, resolved in SDS-PAGE and immunoblotted to assess the expression of stemness associated transcription factors Nanog, Sox-2 and Oct-4. GAPDH was used as a loading control. (**C**) Quantification graphs depict levels of the transcription factors normalized to GAPDH and relative to Vehicle control. Bars represent mean ± SEM of n=3 independent experiments. P values denote a tendency for statistical significance.*

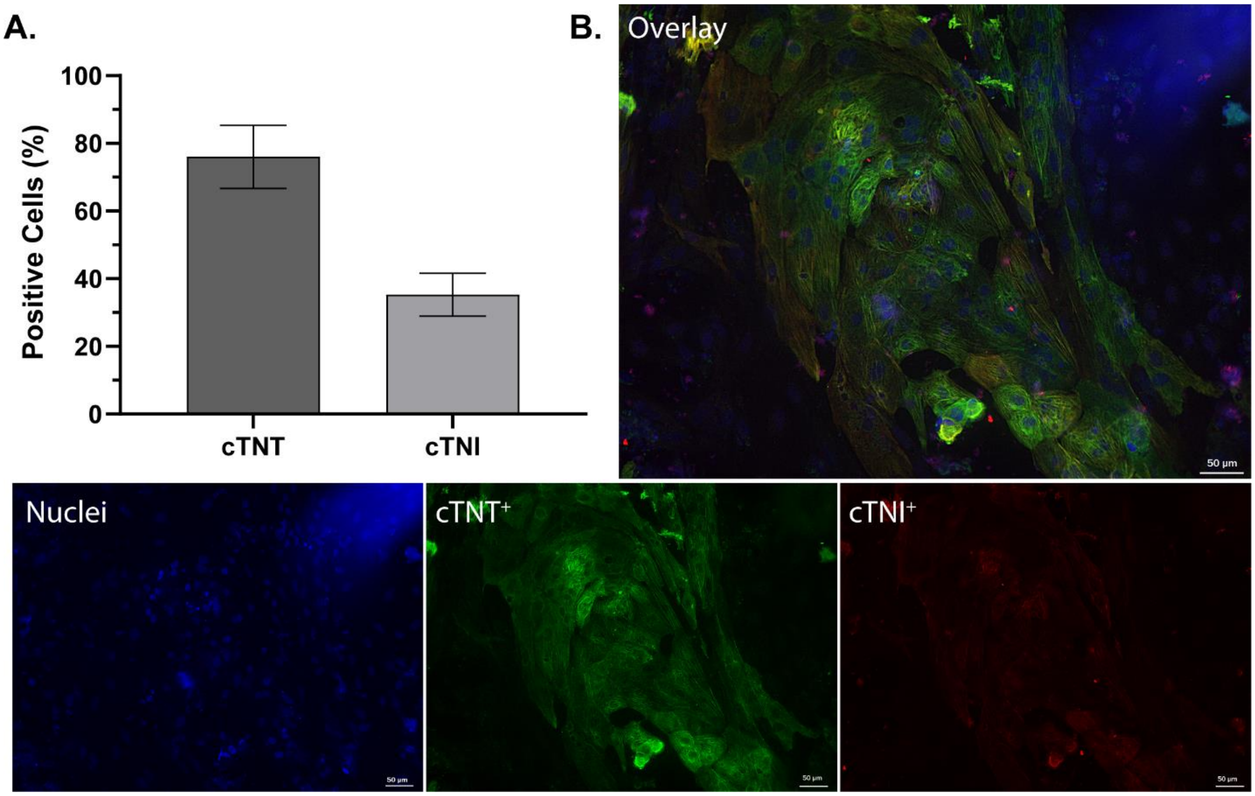

Figure S3. *Cardiac cell biomarkers. (**A**) The number of cardiac biomarkers was measured on 20,000 event using flow cytometry and estimated based on the fraction of cardiac troponin T (cTNT) positive cells. Only cell differentiation with cTNT >70% and cardiac troponin I (cTNI) >30% were considered for this study. Bars represent mean ± SEM of n=6 independent experiments. (**B**) Fluorescent images showing cardiac cell differentiation with channel overlay. Blue indicates nuclear staining (Hoechst), green corresponds to cTNT, and red represents cTNI. The merged image displays the overlay of all three channels. White scale bar = 50 μm.*
